# Aerosolization of a Human Norovirus Surrogate, Bacteriophage MS2, during Simulated Vomiting

**DOI:** 10.1371/journal.pone.0134277

**Published:** 2015-08-19

**Authors:** Grace Tung-Thompson, Dominic A. Libera, Kenneth L. Koch, Francis L. de los Reyes, Lee-Ann Jaykus

**Affiliations:** 1 Department of Food, Bioprocessing and Nutrition Sciences, North Carolina State University, Raleigh, North Carolina, United States of America; 2 Department of Civil, Construction and Environmental Engineering, North Carolina State University, Raleigh, North Carolina, United States of America; 3 Section on Gastroenterology, Wake Forest University School of Medicine, Winston-Salem, North Carolina 27157, United States of America; University of Waterloo, CANADA

## Abstract

Human noroviruses (NoV) are the leading cause of acute gastroenteritis worldwide. Epidemiological studies of outbreaks have suggested that vomiting facilitates transmission of human NoV, but there have been no laboratory-based studies characterizing the degree of NoV release during a vomiting event. The purpose of this work was to demonstrate that virus aerosolization occurs in a simulated vomiting event, and to estimate the amount of virus that is released in those aerosols. A simulated vomiting device was constructed at one-quarter scale of the human body following similitude principles. Simulated vomitus matrices at low (6.24 mPa*s) and high (177.5 mPa*s) viscosities were inoculated with low (10^8^ PFU/mL) and high (10^10^ PFU/mL) concentrations of bacteriophage MS2 and placed in the artificial “stomach” of the device, which was then subjected to scaled physiologically relevant pressures associated with vomiting. Bio aerosols were captured using an SKC Biosampler. In low viscosity artificial vomitus, there were notable differences between recovered aerosolized MS2 as a function of pressure (i.e., greater aerosolization with increased pressure), although this was not always statistically significant. This relationship disappeared when using high viscosity simulated vomitus. The amount of MS2 aerosolized as a percent of total virus “vomited” ranged from 7.2 x 10^-5^ to 2.67 x 10^-2^ (which corresponded to a range of 36 to 13,350 PFU total). To our knowledge, this is the first study to document and measure aerosolization of a NoV surrogate in a similitude-based physical model. This has implications for better understanding the transmission dynamics of human NoV and for risk modeling purposes, both of which can help in designing effective infection control measures.

## Introduction

There are 21 million cases of human norovirus (NoV) infection in the U.S. each year, and this virus genus is now recognized as the leading cause of outbreaks of acute gastroenteritis. According to CDC National Outbreak Reporting Systems (NORS) data for 2009–2010, NoV are responsible for 68% of reported enteric disease outbreaks and 78% of illnesses. They have also been associated with 46% of hospitalizations and 86% of deaths associated with these enteric disease outbreaks. The majority of these outbreaks occur in healthcare facilities (64%), but about 15% occur in association with food service establishments (e.g., restaurants and banquet facilities) [1]. In fact, NoV have been associated for over 50% of all foodborne disease outbreaks [2].

Human NoV can be transmitted by a variety of means. The most widely recognized is the fecal-oral route, which is particularly relevant to contamination of food. However, the virus is also released during projectile vomiting, the hallmark symptom of NoV illness. It is estimated that as many as 30 million virus particles are released in a single episode of vomiting [3,4]. When combined with their low infectious dose (20–1300 particles) [5,6] it is likely that vomiting facilitates NoV transmission. In fact, there have been many outbreaks occurring in hotels, schools, aircraft, concert halls, and cruise ships for which vomiting has been implicated as having a role in transmission [7–11].

Epidemiological evidence from outbreaks suggests that projectile vomiting produces aerosols that contain human NoV [12,13]. Aerosolization of virus during vomiting could potentially extend the spread of virus, result in contamination of surfaces and other fomites, and increase the duration of exposure if viruses remain airborne. Air currents could further disperse aerosolized virus, making contamination even more widespread [4].

Aside from epidemiological studies, the relative importance of aerosol formation in the transmission of human NoV through vomiting is largely unknown. However, there have been studies on aerosolization of influenza virus, usually in association with the physical act of coughing or sneezing. The aerosolization of pathogens by sneezing, coughing, talking, or exhaling depends on many factors such as the flow rate of air suspending the pathogens, evaporation, and the velocity of coughing or sneezing [14,15]. The likelihood of transmission via aerosols is also influenced by the size of the particles, which depends on evaporation, virus aggregation, and properties of the suspending matrix [16].

There are many challenges that hinder work with human NoV, not the least of which is that they cannot be cultivated *in vitro*, nor is there an animal model for their propagation. Consequently, surrogate viruses that are morphologically similar, but cultivable, are often used in studies to mimic human NoV behavior. The male-specific bacteriophage MS2 is one such surrogate that resembles human NoV in that it has a positive sense single stranded RNA genome, icosahedral capsid symmetry, and is within the same size range [17,18]. Bacteriophage MS2 is easily cultivated in the laboratory to high titers (~10^11^ plaque forming units (PFU)/mL). As a bacteriophage, it is also non-pathogenic to humans or animals and is commonly used in aerosolization studies as a surrogate for pathogenic viruses [19].

The purpose of this study was to demonstrate that virus aerosolization occurs in a simulated vomiting event, and to estimate the amount of virus that is released in those aerosols. The work was performed in two parts: (i) creation of a laboratory physical model to simulate human vomiting; and (ii) using that model to characterize the degree of virus aerosolization under various conditions of volume and pressure. The human NoV surrogate MS2 was used in the simulated vomiting experiments.

## Materials and Methods

### Physiological parameters used in vomiting device

To better understand the physiology of vomiting and potential effects on aerosolization, and in the absence of data in the literature, an expert in gastroenterology (author KLK) provided advice to aid in estimating values for key design features. There were a number of parameters in which this advice was useful. The first was volume of vomitus, which varies depending on a person’s height, weight, and diet consumed prior to a vomiting episode. Considering these variations, 800 mL of vomitus in a single vomiting episode was estimated to be a maximum volume. It was assumed to be unlikely that a person would expel less than 50 mL, as this volume might be considered a “dry heave.” The 800 mL estimated vomitus volume was used exclusively in this study to allow the use of a manageable scaled down volume (see Simulated Vomiting Experiments).

The second variable for expert consideration was vomitus viscosity, which depends upon the mix of solid, semi-solid, and liquefied (triturated) foods present in the stomach prior to the vomiting episode. Vomitus with high solids contents would be thick with suspended food particles; pre-gelatinized starch was chosen as a model for high solids content vomitus. Vomitus with low solids contents would be very thin and watery; artificial saliva was used as a model for low solids content vomitus.

It was pointed out that air is present in the gastric fundus, the portion of the stomach immediately distal to the lower esophageal sphincter (LES). The fundic air volume likely contributes to the aerosolization of vomitus and was estimated to be in the range of 50–200 mL. Further, the LES is normally contracted to prevent reflux of gastric content into the esophagus. The normal sphincter pressure ranges from 13 mmHg to 43 mmHg. Increases in intragastric pressure and reverse peristalsis in the gastric antrum and corpus result in abrupt relaxation of the lower esophageal sphincter pressure during vomiting [20]. The upper esophageal sphincter also relaxes during vomiting. Greater intra-abdominal and intra-gastric pressures during vomiting result in more vigorous expulsion of gastric contents and result in the so-called “projectile” vomiting.

Finally, during vomiting the neck is flexed with the mouth pointed toward the ground, a posture that limits the potential for aspiration of vomitus. It was assumed that reproduction of the exact size and shape of the stomach was not necessary in model design as long as scaled lengths and diameters of the esophagus and mouth were used, as well as physiologically relevant pressures of the stomach and esophagus.

### Model Construction

The simulated vomiting device was constructed based on the concept of similitude, which allows a scaled prototype to behave similarly to the full-scale phenomenon being simulated. In this case, the device was designed to function similarly to the full-scale human upper gastrointestinal tract but created at one-quarter scale. Achieving similitude in an engineered model is based on three types of similarity to the full-scale application: geometric, kinematic, and dynamic [21]. Having geometric similarity means that the model and prototype must have the same shape, and that all of the linear dimensions of the model must be related to corresponding dimensions in the prototype by the same scaling factor [21]. To achieve kinematic similarity, velocities at corresponding points in the model must have the same direction and differ by the same constant scale factor as the prototype [21]. Dynamic similarity means that the ratios of all the forces acting on the fluid particles are constant when comparing the model and the prototype. A list of all parameters, data upon which they are based [22–24], their assumptions, and relevant formulas are provided in Table 1. A detailed description of the calculations used for scale up is provided in the Supporting Information (S1 File).

**Table 1:** Summary of Model Parameters, Assumptions, and Relevant Formulae used in Scaling the Simulated Vomiting Device.

	Human Dimension (cm)	Equation Used in Scaling	Machine Dimensions (cm)	Adjusted for Material Availability (cm)
Esophagus Length	25	1	6.35	-
Esophagus Diameter	2.5	1	0.63	-
Mouth Length	9.7	1	2.46	2.54
Mouth Diameter	5.72	1	1.45	1.27
Maximum Vomitus Volume Used	800	2	13.08	-
Minimum Vomitus Volume Used	200	2	3.27	-
Volume of Air in Stomach Used	200	2	3.27	-
Maximum Stomach Pressure	5.6	3	86.8	-
Average Stomach Pressure	1.6	3	24.8	-
Minimum Stomach Pressure	0.77	2	11.9	-
Equations:				
$\left(1\right)\;Machine\;Length=\frac{Human\;Length}{3.94}\;\left(2\right)\;Machine\;Volume=\frac{Human\;Volume}{61.16}\;\left(3\right)\;Machine\;Pressure=Human\;Pressure\;*\;15.5$				

Assumptions:

(1) Flow through the human esophagus and machine esophagus was treated as flow through a smooth pipe.

(2) In some cases, the machine dimensions were rounded to the nearest available dimension offered by material manufacturers.

(3) The vomitus fluid inside the human body will be the same inside the vomiting machine; achieved by using surrogate vomitus with similar viscosities.

**Fig 1:**
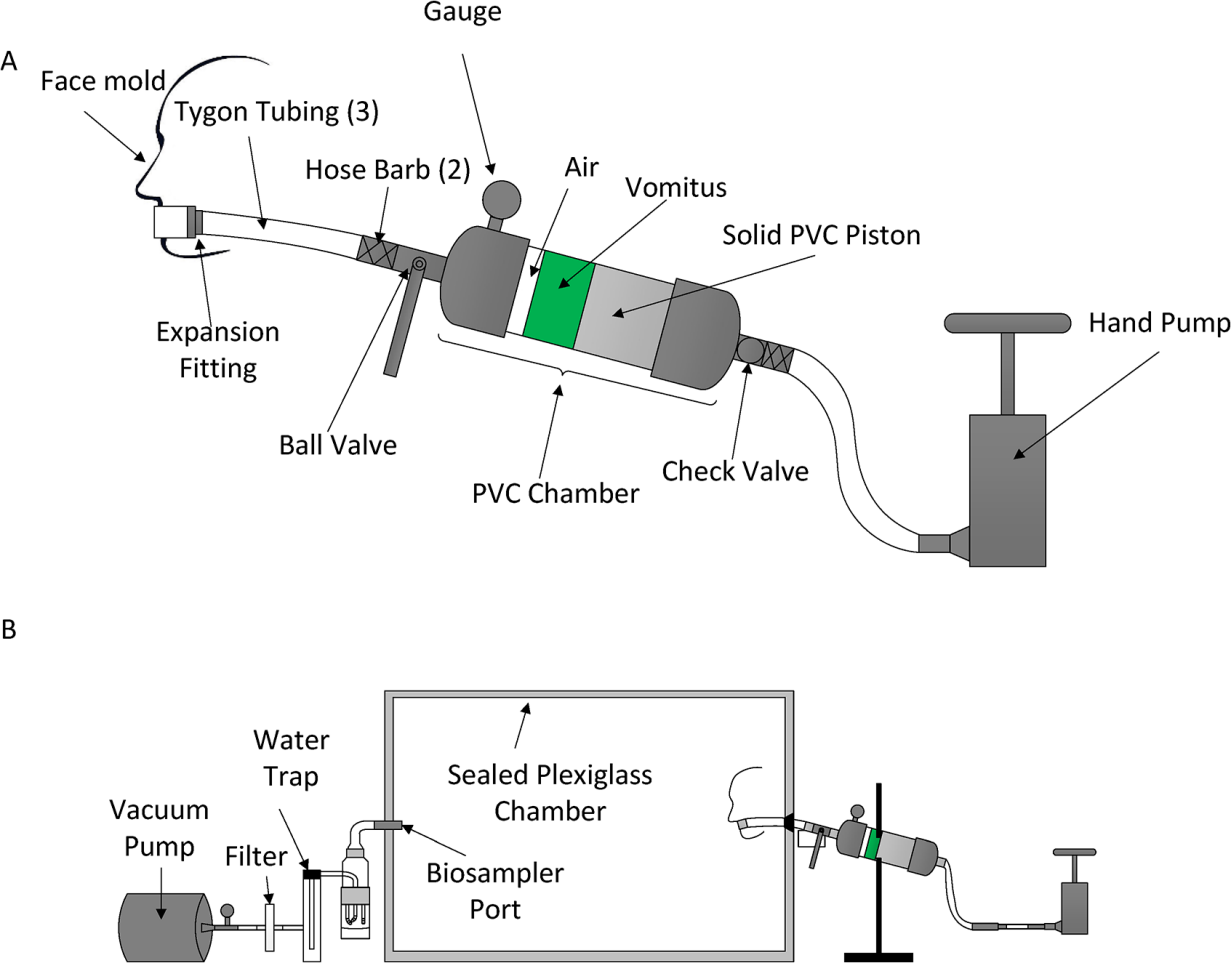
Schematic of Simulated Vomiting Device. **(**A) Diagram of the simulated vomiting device (B) Experimental set-up for capturing aerosolized virus.

Fig 1A shows a diagram of the device. A clear PVC tube (7.62 cm long) attached to two PVC caps, a solid PVC piston, and pressure gauge connected to a pump were designed to act as a surrogate stomach. A brass check valve in the center of one of the PVC caps prevented air from escaping when pressurizing the system. Connecting the stomach chamber to the surrogate esophagus is a ball valve representing the lower esophageal sphincter (LES); in the human body the LES abruptly relaxes in order for the vomitus to be ejected from the stomach. When the valve was opened, the PVC piston pushed the vomitus out of the stomach into the esophagus, represented as a 0.64 cm diameter tube that is 6.35 cm in length. The esophagus was attached to a 1.27 cm diameter tube, representing the mouth, with an expansion fitting. In the device set-up, a slight curve (flexion) was designed in the upper esophagus and “throat” to simulate the flexion of the neck during a vomiting episode. A pressure gauge was attached to the top of the PVC cap to monitor the pressure at the connection between the esophagus and stomach chamber. The ball valve, representing the LES pressure, was opened when the desired intragastric pressure was reached, allowing the PVC piston to eject the vomitus with some velocity out of the stomach and into the esophagus and mouth.

The vomitus containment chamber (Fig 1B) was a Plexiglas box with dimensions of 30.5 cm x 30.5 cm x 44.5 cm and a hinged lid The edges were sealed with weather proofing tape to ensure a tight seal and prevent aerosols from escaping the chamber. On one side of the chamber, the vomiting device was connected to the “vomiting device port”, while on the other side, an SKC© Biosampler (SKC Inc., Eighty Four, PA) was connected to the “biosampler port”. After a simulated vomiting incident, a vacuum pump was operated at a flow rate of 12.5 L/min to facilitate capture of aerosolized particles by the biosampler into 4 mL of phosphate buffered saline (PBS). Preliminary studies indicated that the average biosampler efficiency at capturing aerosolized MS2 was 8.5% (data not shown). The biosampler was run for 15 min (221 chamber volumes) after the simulated vomiting event. The entire set-up was further contained in a Biosafety level II hood.

### Virus Propagation and Enumeration

Bacteriophage MS2 (ATCC 15597-B1) and its *Escherichia coli* C3000 host (ATCC B-15597) were purchased from the American Type Culture Collection (ATCC, Manassas, VA). To prepare MS2 stock solutions, the protocol described in NSF Standard 55 was used (double agar layer method, described below) [25]. After 10-fold serial dilutions of MS2 were plated, those plates showing complete lysis were flooded with 3 mL of tryptic soy broth (TSB) (Fisher Scientific, Pittsburgh, PA) and the soft agar layer was scraped off into a sterile 50 mL tube. The volume was increased to 40 mL with TSB and then 0.2 g EDTA (Sigma-Aldrich, St. Louis, MO) and 0.026 g lysozyme (Fisher Scientific) were added to each tube. The tubes were then incubated for 2 h at 37°C with shaking. The supernatant was recovered by centrifugation at 9,300 *x g* for 10 min followed by filter sterilization using a 0.22 μm filter (Nalgene, Rochester, NY). Aliquots of this were considered high titer MS2 stock (10^10^ PFU/mL). The low titer stock (10^8^ PFU/mL) was prepared by dilution. Stocks were aliquoted and stored at -80°C until use.

Enumeration of MS2 was also performed using the double agar layer method in accordance with the method of Su and D’Souza (2011) with minor modifications [26]. Briefly, the *E*. *coli* C3000 host was incubated for 4–6 h with gentle shaking (100 RPM, 37°C, Excella E24 Incubator, New Brunswick Scientific/Eppendorf, Enfield, CT). Simultaneously, 8 mL tubes of 0.6% tryptic soy agar (TSA) (Fisher Scientific) were melted and tempered in a 42°C water bath. Previously prepared petri dishes (Fisher Scientific) containing 1.2% TSA were allowed to warm to room temperature. Then, 10-fold serial dilutions of MS2 (dilutions to achieve countable plates were as high as 10^−10^ for high titer MS2, 10^−8^ for low titer MS2) were prepared. A volume of 0.7 mL of each dilution was added to the tempered 8 mL TSA tube after which 0.3 mL of *E*. *coli* solution was added, the suspension quickly vortexed and poured on top of the 1.2% TSA plates. Duplicates were done for each dilution. Upon solidification, the plates were inverted, incubated overnight at 37°C and then plaques were counted. Counts were expressed as plaque forming units per milliliter (PFU/mL).

### Simulated Vomiting Experiments

Vomitus solutions consisted of MS2 bacteriophage at high (10^10^ PFU/mL) and low (10^8^ PFU/mL) titer were adjusted to high or low viscosity. To prepare the MS2 low viscosity solution (0.1% carboxymethylcellulose (CMC)), 15 mL of MS2 stock was mixed with 0.15 g of high viscosity CMC powder (pre-hydrated Ticalose CMC 6000 powder; Tic Gums, White Marsh, MD). This was used to simulate artificial saliva with a viscosity of 6.24 mPa*s [27]. For the high viscosity vomitus solution (similar to that of egg yolk), a solution of 25% pre-gelatinized starch (PS) was used. To prepare this, 3.75 g of PS (Vanilla flavor Instant pudding, Jell-O, Glenview, IL) was mixed with 15 mL of MS2 stock. Instant pudding was chosen after consultation with Dr. Tyre Lanier (Department of Food, Bioprocessing and Nutrition Sciences, NCSU) because it was easily attainable and did not affect MS2 viability (data not shown). The solution of PS had a viscosity of 177.5 mPa*s. Solutions exceeding this viscosity were too thick to use in the simulated vomiting device.

A total of 13.1 mL (representing a scaled down volume for 800 mL of vomitus) of each solution was pipetted into the stomach chamber of the device, which contained 3.27 mL of air (scaled down from 200 mL in the human body). Using the pump, the stomach was pressurized to 1,283 mmHg (scaled average pressure experienced in the stomach during projectile vomiting), 290 mmHg (scaled maximum pressure experienced in the human stomach), and 115.1 mmHg (minimum pressure for the device) [24]. Pressures greater than 1,283 mmHg were not used because these approached the pressure gauge capacity. Also, the scaled average pressure in the stomach during projectile vomiting (1,283 mmHg) produced a projectile with a force that appeared to be greater than what would be anticipated in a normal vomiting incident. Therefore, the maximum actual pressure observed in the human stomach (290 mmHg) was assumed to be more relevant and used for comparison purposes. Video observation of recorded human vomiting events showed evidence of coughing after the initial vomiting event. The purpose of coughing is to help clear the airway of debris, to prevent aspiration of foreign materials, and to protect the lungs from overextending maximum inspiration [28]. Therefore, a vomiting event followed by four coughs or retches was also simulated using a pressure of 290 mmHg with 4 “coughs” at 233 mmHg each [24].

The components of the vomiting device and chamber were sterilized using 10% bleach for a 5 min exposure followed by rinsing with tap water and wiping with 70% ethanol. The biosampler was autoclaved after each experiment. A negative control with no MS2 was included in all experiments to demonstrate the absence of cross contamination. Immediately before experiments, the entire device was exposed to 254 nm of ultraviolet light for 1 h. Samples collected (by pipet) and analyzed (enumerated for MS2) included: (i) MS2 stock aliquot; (ii) MS2 with thickener (inoculated artificial vomitus solution); (iii) PBS from the biosampler (captured aerosolized virus); (iv) PBS rinse of the biosampler (residual captured aerosolized virus); and (v) liquid splatter on the bottom of the chamber. Volumes of samples collected and amount of surrogate vomitus remaining in the stomach chamber were also recorded. Previous experiments, in which the chamber was swabbed after a simulated vomiting event, and those swabs enumerated for MS2, confirmed that virus deposition on dry surfaces of the chamber was minimal (cumulatively, <0.1% of total input) (data not shown).

### Statistical Analysis

Experiments were performed in triplicate. To calculate the amount of MS2 aerosolized, the concentration of MS2 captured by the biosampler was normalized for both volume and for biosampler capture efficiency (8.5%). The Holm-Sidak multiple comparisons test (SigmaPlot, San Jose, CA) was used for all pairwise comparisons between treatments and pressures. Statistical significance was established at *p*<0.05,**α** = 0.05. For comparing the percent recoveries, the data were not normally distributed; therefore non-parametric Kruskall-Wallis ANOVA by ranks was performed.

### Ethics Statement

This research meets all applicable standards for the ethics of experimentation and research integrity.

## Results

A snapshot of a simulated projectile vomiting event is shown in Fig 2 and a video recording hosted at (http://youtu.be/jGvqb87DXSI). After each vomiting episode, virtually all of the vomitus solution was deposited at the bottom of the chamber. However, there was evidence of aerosolized MS2 after every simulated vomiting episode (Fig 3). At low initial MS2 inoculum titer (10^8^ PFU/mL) suspended in 0.1% CMC (low viscosity), there were statistically significant differences between log_10_ concentration of MS2 recovered from aerosolized vomitus as a function of pressure (i.e., higher MS2 concentration with higher pressure). The same occurred for high titer (10^10^ PFU/mL), low viscosity experiments, although these differences were not statistically significant. There appeared to be little relationship between pressure and aerosolization for the high titer, high viscosity experiments. The high viscosity, high titer MS2 inoculum was not expelled out of the simulator at the low pressure of 115.1 mmHg, presumably because of the low expulsion force. There was also no statistically significant difference in the concentration of MS2 aerosolized for high viscosity, high titer MS2 solution when compared to low viscosity, high titer MS2 simulated vomitus solution. Although not statistically significant, there appeared to be a slight difference of increased MS2 aerosolization when simulated coughing (at 290 mmHg) was added, regardless of virus titer or simulated vomitus viscosity.

**Fig 2:**
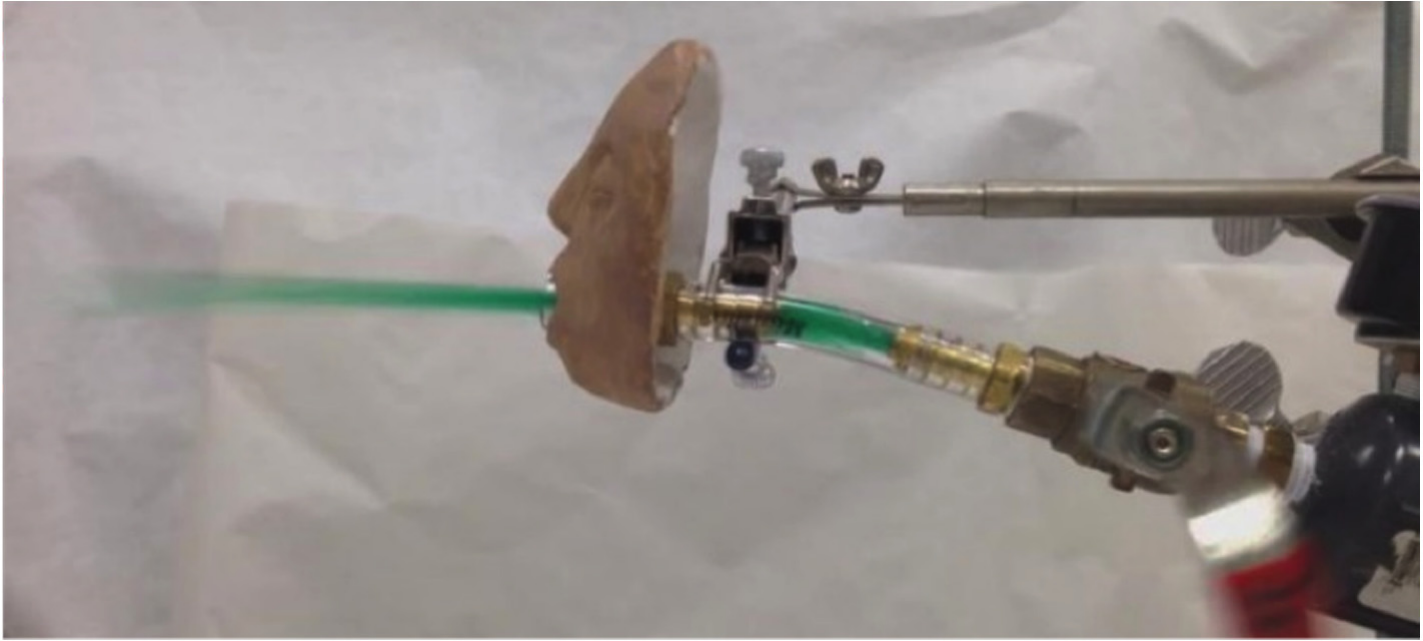
Photo of a Simulated Vomiting Episode. Projectile vomiting of colored simulated vomitus matrix.

**Fig 3:**
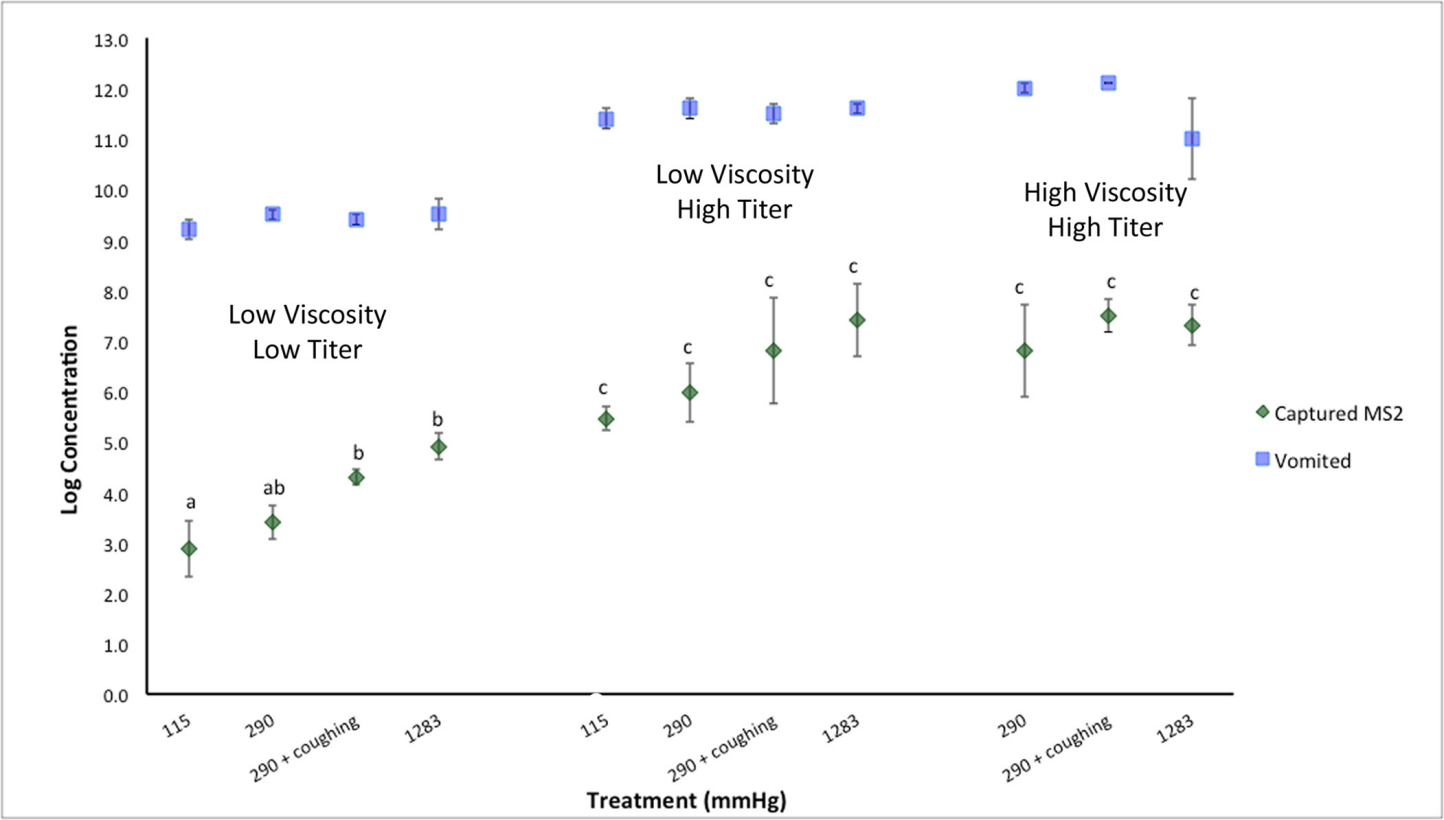
Aerosolization Experiments using bacteriophage MS2. Virus concentration “vomited” is designated by blue squares. Green diamonds show the amount of captured MS2 at designated pressures for simulated vomitus having low and high MS2 titer and of low and high viscosity. Error bars denote one standard deviation above the mean. Shared letters and symbols indicate treatments that were not statistically significantly different within each group.

The amount of MS2 aerosolized as a percent of total virus “vomited” ranged from a low of 7.2 x 10^−5^ ± 0.00006 to a high of 2.67 x 10^−2^ ± 0.03 (Table 2). These data were not normally distributed; therefore non-parametric Kruskall-Wallis ANOVA by ranks was performed. There were statistically significant differences between vomiting conditions and degree (%) of MS2 aerosolization (*p*<0.01). When the data were log-transformed and reanalyzed, there were no statistically significant differences when comparing MS2 percent aerosolization at 1,283 mmHg to 290 mmHg with coughing, regardless of virus titer or solution viscosity. There were statistically significant differences when comparing percent aerosolization at pressures of 1,283 mmHg and 115 mmHg (*p*<0.05). The general trend was greater percent aerosolization for high titer MS2 at 1,283 mmHg and 290 mmHg with coughing, than 290 mmHg and 115 mmHg without coughing.

**Table 2:** Percent Recoveries of Aerosolized MS2.

Treatment		% Aerosolized	Log % Aerosolized	Statistical Significance			
1,283 mmHg	Low Viscosity, Low Titer	2.8 x 10^−3^ ± 0.001	-2.58 ± 0.21	A	B	C	
	Low Viscosity, High Titer	1.3 x 10^−2^ ± 0.01	-2.2 ± 0.81	A	B		
	High Viscosity, High Titer	2.7 x 10^−2^ ± 0.03	-1.72 ± 0.42	A			
290 mmHg	Low Viscosity, Low Titer	1.1 x 10^−4^ ± 0.00005	-4.02 ± 0.24			C	D
	Low Viscosity, High Titer	4.6 x 10^−4^ ± 0.0005	-3.58 ± 0.63		B	C	D
	High Viscosity, High Titer	1.4 x 10^−3^ ± 0.001	-3.29 ± 1.00	A	B	C	D
290 mmHg + coughing	Low Viscosity, Low Titer	9.6 x 10^−4^ ± 0.0005	-3.06 ± 0.24	A	B	C	D
	Low Viscosity, High Titer	1.1 x 10^−2^ ± 0.02	-2.55 ± 0.93	A	B	C	
	High Viscosity, High Titer	3.2 x 10^−3^ ± 0.002	-2.57 ± 0.31	A	B	C	
115 mmHg	Low Viscosity, Low Titer	7.2 x 10^−5^ ± 0.00006	-4.35 ± 0.63				D
	Low Viscosity, High Titer	1.33 x 10^−4^ ± 0.00009	-3.93 ± 0.27		B	C	D
						

* Shared letters denote treatments with no statistically significant differences at *p*>0.05.

## Discussion and Conclusions

By simulating vomiting using a device scaled to human physiological parameters according to similitude principles, this study demonstrated that virus (MS2) aerosolization did indeed occur. These results complement the recent work of Bonifait et al. (2015), who provided the first definitive evidence of NoV bioaerosolization [29]. Specifically, they found evidence of NoV genogroup II in 8/48 air samples collected; positive samples had concentrations (by RT-qPCR) of 1.4 x 10^1^–2.4 x 10^3^ genome copies per m^3^ of air. We, on the other hand, provide evidence that virus aerosols can be produced during the act of vomiting. Together, our work and that of Bonifait et al. (2015) add to the growing evidence that NoV aerosolization occurs by vomiting.

In all cases, <0.03% of the initial concentration of MS2 in the artificial vomitus was aerosolized, though the numbers were quite variable. While a small percentage of the virus released during simulated vomiting was aerosolized, what was released could be enough to cause a significant disease risk. For instance, if an individual vomits at least 50 mL with at least 10^6^ particles/mL (numbers from Greenberg et al. (1979)), this would mean 5 x 10^7^ particles would be vomited. Even with the lowest percent of aerosolized virus (7.2 x 10^−5^% for 115 mmHg at low titer), approximately 36 virus particles would become aerosolized. In contrast, the highest percent aerosolized (2.67 x 10^−2^% shown at 1,283 mmHg for high titer, high viscosity artificial vomitus) would result in aerosolization of >13,000 particles. Interestingly, these numbers are consistent with those estimated for bioaerosols in outbreak settings (1.4 x 10^1^–2.4 x 10^3^ genome copies per m^3^ of air) by Bonifait et al. (2015)[29]. Given the low infectious dose of human NoV (20–1300 particles) [5,6], these numbers are clearly enough to make exposed susceptible individuals ill.

Spatial associations and attack rate patterns occurring as a consequence of vomiting incidents support human NoV aerosolization. Marks et al. (2000) demonstrated that attack rates were related to how far individuals sat from the initial vomiting incident in a hotel restaurant: 91% for those sitting at the same table, 56–71% for those at adjacent tables, and 25% for those seated at the table furthest from the incident [12]. Similarly, Harris et al. (2013) showed that individuals in the same vicinity within a hospital as patients with symptoms of human NoV infection were more likely to become infected than individuals further away [30]. Such spatial associations may be a function of the number and droplet size during vomiting. Smaller droplets may remain in the air for longer periods of time and be subjected to indoor air movements, thus traveling further. On the other hand, larger droplets would be more likely to settle to the surface closer to the initial vomiting incident [31,32].

Pressure was a major parameter investigated in this study. Booth (2014) recently reported on a simulated vomiting system that was used to characterize the extent of splatter occurring in vomiting event [33]. Although that study did not examine aerosolization, an authentic mannequin that is typically used for training adult airway management, with realistic anatomy parts of the upper respiratory tract and an esophagus and stomach (which was replaced with a cylinder containing 1 L of fluid) was used. That study reported that, for their model, a pressure of 6,000 mmHg was required to eject 1 L of water a distance of 1.2 meters. This pressure is significantly greater than the 1,283 mmHg maximum pressure that we used in our simulated vomiting experiments, which was scaled to the average pressure in the human stomach during a vomiting incident as reported by Iqbal et al., 2008 [24]. Assuming that the Booth model was exactly human scale, the pressures required to model vomiting are almost 20 times greater than the average values reported by Iqbal et al., 2008 [24].

Although the amount of virus aerosolized was generally positively correlated with the pressure with which the vomitus was released, this relationship was not always statistically significant. This was partially due to the large standard deviations in the measurements, suggesting high variability in degree of virus aerosolization during vomiting. This implies that even a relatively minor vomiting event may have public health significance. We did not observe a major role for viscosity in the degree of virus aerosolization, despite the fact that others have found that suspension media can play an important role in resistance of virus to aerosolization [19].

Human NoV particles have a diameter of 32 nm and a buoyant density of 1.41 g/cm^3^ [12]. Particles this small undergo random Brownian motion and will eventually collide with other particles and coagulate to form larger particles. Based on the parameters above, the settling velocity for a single NoV particle, calculated using Stokes’ law, is 4.7 x 10^−8^ m/s. This is very slow, and if left uninterrupted, the virus could remain in the air for months. Of course, it is highly unlikely that virus travel would remain uninterrupted, or that single viruses would be aerosolized without some attachment to the suspending matrix.

Hence, droplets formed as a consequence of a vomiting incident are very important in transmission. Droplet transmission occurs when aerosolized particles are large enough (100–500 μm in diameter) to settle to the ground quickly. For example, a 100 μm droplet, with density of 1.41 g/cm^3^ settling in air at 20° C, is predicted to travel 0.46 m/s, meaning that for a distance of 1 meter it will only take the droplet a few seconds to reach the ground [34]. These droplets can also fall on inanimate surfaces, resulting in contamination of fomites.

Consistent with the work of others [35–37], we used the SKC Biosampler for quantifying virus recovery due to aerosolization. This biosampler has been shown to be better for retaining virus infectivity [35], and in comparative studies with other biosamplers, has also been found to be the most efficient at virus capture [19,35,36]. However, there is wide variability in the reported efficiency of virus capture using the SKC Biosampler. For example, Fabian et al., (2009) reported 96% collection efficiency for aerosolized influenza virus particles >1 μm in diameter, and 79% for particles 0.3 μm in diameter using the SKC unit [36]. Others have reported lower capture efficiencies. Hogan et al., 2005 demonstrated <10% efficiency for capturing aerosolized MS2 particles of 30–100 nm in diameter using the SKC Biosampler [37], while Turgeon et al., 2014 found MS2 recovery to be approximately 0.1% as determined by plaque assay and qPCR [19]. We observed recovery efficiencies similar to these (8.5%) when a nebulizer was used to aerosolize MS2 with the SKC Biosampler.

Although due diligence was taken in model and experimental design, there are a few limitations to this study. For instance, even though the equipment was appropriately scaled, the structural features of the simulated vomiting device were not the same as human anatomy. While the pressures used for simulated vomiting were scaled, the force with which the vomiting occurred using the device sometimes appeared greater than one might expect in real life. Vomitus in nature would undoubtedly contain solids, and the use of solids-free simulated fluids could have impacted the likelihood or degree of virus aerosolization. Based on the model’s design, a solids-containing suspension could not be used. The SKC Biosampler has been shown to be more effective at collecting larger airborne particles, but is unable to distinguish particle size. There may potentially be greater aerosolization that could not be detected using the SKC Biosampler, as the efficiency of recovery decreased as size of the particle decreased. Lastly, although MS2 is a logical surrogate virus for human NoV because of its ease of enumeration and safety, it is still necessary to extrapolate the behavior of the surrogate to that of human NoV. Not only has MS2 been a popular surrogate for many pathogenic viruses, it is often used in aerosol studies to examine air samplers and aerosol generation techniques [37,38]. MS2 is also environmentally persistent, like human NoV [39]. Surrogate viruses, like MS2, have been used in other virus aerosolization studies [40–42]. We note that the experimental approach used here, and employing a physical model designed according to similitude principles, may be useful in studies of aerosolization of other viruses during vomiting. For those studies, other surrogate viruses that are similar to size, composition (e.g., lipid envelop or non-enveloped), and other characteristics to the virus being modeled, would be more appropriate.

To our knowledge, this is the first study to document and measure aerosolization of a NoV surrogate in a similitude-based physical model. Relative to the MS2 titers “vomited,” the degree of aerosolization was rather minimal (<0.01%). However, based on human NoV infectious dose and estimated virus concentrations in vomitus, even these small percentages of aerosolization would likely result in significant disease risk, as was suggested in the recent findings of Bonifait et al. (2015) [29]. Future studies should focus on characterizing aerosolized particle droplet size as this plays an important role in the settling rate of viruses. The work reported here has implications for better understanding the transmission dynamics of human NoV and for risk modeling purposes, both of which can help in designing effective infection control measures.

## Supporting Information

S1 File Supplementary Material Explaining Concepts and Equations for the Construction of the Vomiting Machine. (PDF)
