# Supplementary material for: Aerosolization of a Human Norovirus Surrogate, Bacteriophage MS2, during Simulated Vomiting
Source: PLoS One. 2015 Aug 19;10(8):e0134277. doi: 10.1371/journal.pone.0134277 (PMC4545942; doi:10.1371/journal.pone.0134277)
Supplement: S1 File — (PDF) [file pone.0134277.s001.pdf]

Supplementary Material

This supplementary material describes the concepts and the derivations of equations used to construct the laboratory vomiting machine. A human full-scale model was not feasible to maintain in a sterile lab environment, and similitude was used to produce a scaled version of the model.

Similitude Overview

Similitude is a concept in fluid mechanics that is used to make a scaled engineering model similar to a full-scale prototype. In this project the full- scale prototype is defined as the human upper gastrointestinal tract including the mouth, esophagus, and stomach. The full-scale prototype will be referred to as the "human body" and/or have an "h" subscript while the scaled model with have an "m" subscript. Achieving similitude in an engineering model is based on three types of similarity to the full-scale application: geometric, kinematic, and dynamic (Fox Robert, McDonald Alan, & Pritchard Philip, 2004). Having geometric similarity in an engineering model means that the model and prototype must have the same shape and that all of the linear dimensions of the model must be related to corresponding dimensions in the prototype by the same scaling factor. A requirement for kinematic similarity is that velocities at corresponding points in the model and prototype must have the same direction and differ by the same constant scale factor. Dynamic similarity means that the ratios of all the forces acting on the fluid particles are constant between the engineering model and the prototype. To achieve dynamic similarity certain dimensionless groups must have the same value in the model and in the prototype. We used the Buckingham Pi theorem to identify the dimensionless groups, also known as π groups, appropriate for the vomiting problem (Fox Robert et al., 2004).

Determining π Groups

The first step in determining the π groups is to list all the dimensional parameters that affect fluid flow in the fluid mechanics problem; the number of dimensional parameters in this list is denoted by the term *n*. Flow through the human body esophagus and scaled model esophagus was treated as flow through a smooth pipe. Dimensional parameters that affect fluid flow through a smooth pipe are: Δp (pressure change), D (pipe diameter), V (fluid velocity), ρ (fluid density), and μ (fluid dynamic viscosity); thus *n* = 5 (Munson & Okiishi, 2002).

The second step is to list all of the primary dimensions that are found in the dimensional parameters; the number of primary dimensions is noted by the term *m*. The primary dimensions found in the dimensional parameters include: M (mass), L (length), and T (time); thus *m* = 3. The dimensional parameters are listed below followed by the form of their primary dimensions in parentheses: $\Delta p\ \left(\frac{M}{T^2L}\right)$, $D\ (L)$, $V\ \left(\frac{L}{T}\right)$, $\rho\ \left(\frac{M}{L^3}\right)$, $\mu\ \left(\frac{M}{LT}\right)$.

The third step is to select a group of repeating dimensional parameters that will appear in all of the π groups for subsequent steps. One requirement for a repeating dimensional parameter is that it cannot have dimensions that are a power of another dimensional parameter. For instance, in this situation Δp and μ cannot be repeating dimensional parameters because they have dimensions $(MT^{-2}L^{-1})$ and $(MT^{-1}L^{-1})$, respectively. Thus D, V and ρ are repeating dimensional parameters. Next, the Buckingham π theorem is used to determine the number of π groups needed for dynamic similarity. The theorem states that *n-m* dimensionless groups are needed; in this case two π groups are needed for this fluid mechanics problem.

The 1st π group should follow the functional form shown in Equation S.1, and include all of the repeating dimensional parameters and one of the two non-repeating dimensional parameters. Equation S.2 lists the elements of the 1st π group in terms of its dimensions; for this

equation to be dimensionless, Equation S.2 must hold true for the values of the exponents: $a, b, and\ c$.

$$\pi_1 = \Delta p \cdot D^a \cdot V^b \cdot \rho^c \tag{S.1}$$

$$\pi_1 = \left(\frac{\mathrm{M}}{\mathrm{T}^2\mathrm{L}}\right)\cdot(\mathrm{L})^a \cdot \left(\frac{\mathrm{L}}{\mathrm{T}}\right)^b \cdot \left(\frac{\mathrm{M}}{\mathrm{L}^3}\right)^c = \mathrm{M}^0 \cdot \mathrm{L}^0 \cdot \mathrm{T}^0 \tag{S.2}$$

$$M\,balance: 1 + c = 0 \tag{S.3}$$

$$T\,balance: -2 - b = 0 \tag{S.4}$$

$$L\ balance: -1 + a + b - 3c = 0 \tag{S.5}$$

To solve for the exponents in Equation S.2, each dimension is balanced on both sides of the equation as shown in Equations S.3, S.4, and S.5. Solving the equations simultaneously yields the following values: $c = -1, b = -2, a = 0$. Substituting the exponents back into Equation S.1 yields the 1st π group, shown in Equation S.6.

$$\pi_1 = \frac{\Delta p}{V^2 \cdot \rho} \tag{S.6}$$

The 2nd π group includes all of the repeating dimensional parameters and μ, the second of the two non-repeating dimensional parameters, seen in Equation S.7. For the 2nd π group to be dimensionless, Equation S.8 must hold true for the values of $d, e, and\ f$.

$$\pi_2 = \mu \cdot D^d \cdot V^e \cdot \rho^f \tag{S.7}$$

$$\pi_2 = \left(\frac{M}{L \cdot T}\right)\cdot(L)^d \cdot \left(\frac{L}{T}\right)^e \cdot \left(\frac{M}{L^3}\right)^f = M^0 \cdot L^0 \cdot T^0 \tag{S.8}$$

$$M\,balance: 1 + d = 0 \tag{S.9}$$

$$T\ balance: -1 - e = 0 \tag{S.10}$$

$$L\ balance: -1 + d + e - 3f = 0 \tag{S.11}$$

To solve for the exponents of the dimensional parameters in Equation S.8, each dimension is balanced on both sides of the equation as shown in Equations S.9, S.10 and S.11. Solving the equations simultaneously yields the following values: $d = -1, e = -1, f = -1$. Substituting the exponents into Equation S.7 yields the 2nd π group, shown in Equation S.12.

$$\pi_2 = \frac{\mu}{D \cdot V \cdot \rho} \tag{S.12}$$

Recall that dynamic similarity is achieved when the π groups have the same value in the model and in the human body. Note that the 1st π group is closely related to a common dimensionless parameter used in fluid mechanics, known as the Euler number or pressure coefficient. The relationship between the 1st π group and the pressure coefficient is shown in Equation S.13. Additionally, the 2nd π group is similar to a common dimensionless parameter known as the Reynolds number; its relationship is shown in Equation S.14 (Fox Robert et al., 2004).

$$\pi_1 = \frac{1}{2} \cdot (C_p) \tag{S.13}$$

$$\pi_2 = (\mathrm{Re})^{-1} \tag{S.14}$$

Since the Reynolds number and pressure coefficient are common dimensionless parameters and are easy to work with mathematically, they will be used as the dimensionless groups to achieve dynamic similarity in the scaled model (Fox Robert et al., 2004). Equations S.15 and S.16 show that the Reynolds number and pressure coefficient must be the same for both the scaled model (denoted by the *m* subscript) and the human body (denoted by the *h* subscript).

$$Re_h = Re_m = \left(\frac{\rho_h D_h V_h}{\mu_h} \right) = \left(\frac{\rho_m D_m V_m}{\mu_m} \right) \tag{S.15}$$

$$Cp_h = Cp_m = \left(\frac{\Delta p_h}{\frac{1}{2} \rho_h V_h^2} \right) = \left(\frac{\Delta p_m}{\frac{1}{2} \rho_m V_m^2} \right) \tag{S.16}$$

Achieving Similitude

Using the dimensions of the human body, outlined in Table 2, several trial-and-error iterations were performed to find a scaling factor that would create the easiest assembly of the model with respect to material availability. Approximately a four to one scale was used to construct the vomiting machine device. Below Equation S.17 shows an example using the linear scaling factor when scaling down the length of a human esophagus, L_h, to the length of the surrogate esophagus in the model, L_m.

$$\text{Linear Scaling Factor} = \frac{L_h}{L_m} = \frac{25\ cm}{6.35\ cm} = 3.94 \tag{S.17}$$

Geometric similarity was achieved by scaling down every linear human dimension by the linear scaling factor to be used in model dimensioning, as shown in Equation S.18.

$$\text{Model Length} = \frac{\text{Human Length}}{3.94} \tag{S.18}$$

Shown in Table 2 are the values of all the scaled linear dimensions used in the model found using Equation S.18. Since vomitus volume and volume of air in the stomach are cubic dimensions and not linear dimensions then the scaling linear factor can not be used. To find the cubic scaling factor the linear scaling factor should be cubed; the cubic scaling factor is shown in Equation S.19.

$$\text{Model Volume} = \frac{\text{Human Volume}}{(3.94)^3} = \frac{\text{Human Volume}}{61.16} \quad \text{(S.19)}$$

In some cases, the scaled model dimensions were rounded to the nearest available dimension offered by product manufacturers, as shown in Table 2.

For similitude purposes, an assumption was made that the vomitus fluid inside the scaled model is the same as vomitus inside the human body. Under this assumption, Equation S.15 reduces to Equation S.20.

$$(D_h V_h) = (D_m V_m) \quad \text{(S.20)}$$

Equation S.20 has the term "D" which refers to diameter; since this term is a linear dimension, we know the scaling relationship between D_h and D_m according to Equation S.18. Substituting Equation S.18 into Equation S.20 reveals the constant scale factor for velocities shown in Equation S.21.

$$\frac{V_h}{V_m} = \frac{D_m}{D_h} = \frac{1}{3.94} \quad \text{(S.21)}$$

Velocities of vomitus inside the human body and the scaled model are unknown at this time. Since the specific velocity values in the model and the human body are unknown, Equation S.21 serves primarily as a guide to the ratio of velocities rather than to the specific values of velocities.

The governing dimensional parameter of our scaled model will be pressure. The pressure coefficient will be more of concern than Reynolds number when proving dynamic similarity because the pressure coefficient has a pressure term in its π group. Under the assumption that the

same fluid is used in the model as in the human body, S.16 reduces to Equation S.22. Rearranging Equation S.22 and solving for Δp_m, change in pressure in the model, yields Equation S.23.

$$\frac{\Delta p_h}{V_h^2} = \frac{\Delta p_m}{V_m^2} \tag{S.22}$$

$$\Delta p_m = \Delta p_h \cdot \left(\frac{V_m}{V_h} \right)^2 \tag{S.23}$$

Combining the reduced Reynolds number expression, Equation S.21, into Equation S.23 reveals Equation S.24.

$$\Delta p_m = \Delta p_h \cdot (3.94)^2 \tag{S.24}$$

The change in pressure term, Δp is defined as the difference in local pressure, p minus the freestream pressure, p_∞ as shown in Equation S.25 (Fox Robert et al., 2004). In both the model and the human body, the freestream pressure is zero. The local pressure, p_p, in the human body will be at the point where the esophagus connects to the stomach and the local pressure in the model, p_m, will be at the point where the surrogate esophagus connects to the stomach chamber.

$$\Delta p = p - p_\infty \tag{S.25}$$

Since the freestream pressures are zero, Equation S.25 reduces to Equation S.26.

$$p_m = p_h \cdot (3.94)^2 = p_h \cdot 15.5 \tag{S.26}$$

Dynamic similarity was achieved by scaling down the pressures observed in the human stomach by the pressure scaling factor to be used in model construction, as shown in Equation S.27.

$$\text{Model Pressure} = \text{HumanPressure} \cdot 15.5 \qquad (S.27)$$

References

Fox Robert, W., McDonald Alan, T. & Pritchard Philip, J. (2004). Introduction to Fluid Mechanics. Hoboken, NJ: Wiley & Sons, Inc.

Munson, B. R. D. F. Y. & Okiishi, T. H. (2002). Fundamentals of Fluid Mechanics. *John Wiley & Sons, Inc*.
